# Socially-assigned race and health: a scoping review with global implications for population health equity

**DOI:** 10.1186/s12939-020-1137-5

**Published:** 2020-02-10

**Authors:** Kellee White, Jourdyn A. Lawrence, Nedelina Tchangalova, Shuo J. Huang, Jason L. Cummings

**Affiliations:** 1 Department of Health Policy and Management, University of Maryland College Park School of Public Health, 3310B SPH Bldg 255, 4200 Valley Drive, College Park, MD 20742 USA; 2 Department of Social and Behavioral Sciences, Harvard T.H. Chan School of Public Health, Boston, MA USA; 3 Research and Academic Services, University of Maryland Libraries, College Park, MD USA; 4 Department of Sociology and African American Studies, University of South Carolina, Columbia, SC USA

**Keywords:** Socially-assigned race, Health equity, Racial/ethnic disparities, population health, Scoping review

## Abstract

Self-identified race/ethnicity is largely used to identify, monitor, and examine racial/ethnic inequalities. A growing body of work underscores the need to consider multiple dimensions of race – the social construction of race as a function of appearance, societal interactions, institutional dynamics, stereotypes, and social norms. One such multidimensional measure is socially-assigned race, the perception of one’s race by others, that may serve as the basis for differential or unfair treatment and subsequently lead to deleterious health outcomes. We conducted a scoping review to systematically appraise the socially-assigned race and health literature. A systematic search of the PubMed, Web of Science, 28 EBSCO databases and 24 Proquest databases up to September 2019 was conducted and supplemented by a manual search of reference lists and grey literature. Quantitative and qualitative studies that examined socially-assigned race and health or health-related outcomes were considered for inclusion. Eighteen articles were included in the narrative synthesis. Self-rated health and mental health were among the most frequent outcomes studied. The majority of studies were conducted in the United States, with fewer studies conducted in New Zealand, Canada, and Latin America. While most studies demonstrate a positive association between social assignment as a disadvantaged racial or ethnic group and poorer health, some studies did not document an association. We describe key conceptual and methodological considerations that should be prioritized in future studies examining socially-assigned race and health. Socially-assigned race can provide additional insight into observed differential health outcomes among racial/ethnic groups in racialized societies based upon their lived experiences. Studies incorporating socially-assigned race warrants further investigation and may be leveraged to examine nuanced patterns of racial health advantage and disadvantage.

## Introduction

The most commonly used approach to collect, measure, and analyze race/ethnicity is self-identified (or self-reported) by the respondent. Directives established by the United States Office of Management and Budget (OMB) have mandated the standards and provided guidance for the collection of race and ethnicity data [1–3]. To self-identify race/ethnicity, individuals are asked to respond to two separate questions about Hispanic ethnicity (“Are you of Hispanic, Latino, or Spanish origin?”) and race (“What is your race?”). This method of measuring race/ethnicity has been critical to current knowledge of racial/ethnic differences in education, employment, and health. For example, research has consistently documented variations in morbidity and mortality by self-identified race/ethnicity, noting poorer health outcomes among historically oppressed and underrepresented groups (i.e., Blacks/African Americans, Latinos, and Native Americans) in the United States [4]. Moreover, studies have consistently shown that even after accounting for factors known to influence disease risk such as socioeconomic status, health behaviors and healthcare, self-identified race/ethnicity remains a salient predictor of overall well-being and health. Unsatisfactory progress in efforts to eliminate racial/ethnic health inequities suggest a need for centering racism as a primary mechanism of race-associated differences in health and broadening the conceptualization of race to accurately reflect the lived experience of individuals in a racialized society.

Several scholars contend that self-identified race/ethnicity does not adequately characterize the contextual aspect of race – the lived experience and opportunities of racial/ethnic groups [5–7]. It has been argued that self-identified race/ethnicity is not sufficient to represent the individual and structural components of experiencing race in a racialized society [6, 8]. First, a singular reliance upon self-identified race/ethnicity may conceal intraracial heterogeneity in the experience of race and racism [7, 9]. Members of the same racial/ethnic group may have vastly different lived experiences based on how others perceive them. The social interactions of an individual who self-identifies as black and is perceived as white may be qualitatively different from the social interactions of an individual who self-identifies as black but is perceived as black. For example, individuals who self-identify and are socially-assigned as black may have a higher likelihood of exposure to daily microaggressions and racial discrimination relative to individuals who self-identify as black and socially-assigned as white. Second, there is a growing divergence between how respondents identify and how others see them [7, 10]. For instance, Latinx populations are frequently socially-assigned to a race that is inconsistent with their self-identification [11]. The experience of race is not static and can change for an individual as a function of social relationships, time, and context [8]. The changing population demographics in the United States present new opportunities for understanding the complexity of race and mechanisms that produce and maintain racial inequality in an increasingly multiracial and majority-minority society [12]. Solely relying on self-identified race to measure group membership may not be sufficient to capture the relational nature of race, particularly where racism is the central underlying mechanism.

There is a growing body of literature highlighting the need for a more thoughtful alignment with theoretical work emphasizing the multidimensionality of race [5, 8, 13, 14]. Accounting for multiple dimensions of race describes the ways that race is socially constructed beyond self-identification and is dependent upon external perceptions and classification which shape how an individual is treated. Components of a multidimensional race measure comprise racial self-classification, racial identity, reflected appraisals (e.g., socially-assigned race), phenotype, and racial ancestry [5]. These multidimensional measures may offer a more detailed representation of the relationship between race and health. Measuring the collective impact of race/ethnicity, beyond self-identified race/ethnicity, to include how individuals are perceived and treated by others may facilitate the examination of more nuanced patterns of racial health advantage and disadvantage [15].

One multidimensional measure of race that is increasingly used in the public health literature is socially-assigned (i.e, or ascribed) race, the racial/ethnic categorization of individuals by others. The external classification of race is typically based on physical appearance and phenotypic markers (e.g., skin complexion) that largely reflect how perceptions by the dominant or mainstream social groups. It has been noted that an individual’s racial self-identification may be distinct from how they are seen by others [16]. Socially-assigned race can further our understanding of racial health inequalities via racialization, implicit bias, racial discrimination, and white advantage. Omni and Winant define racialization as the process of attaching racial meaning and value to individuals and groups [17]. Racialization is considered the beginning step in the process of racism [18, 19]. It has been argued that it is the socially-assigned race of an individual, the imposed classification of race by others, that results in racial discrimination more so than how one self-identifies [10, 20]. For example, the external classification of an individual’s race/ethnicity may more accurately reflect the race/ethnicity that is noted in everyday social interactions by a police officer, judge, physician, teacher, hiring manager, or a sales clerk [20]. The inherent negative value assigned to one’s race via explicit, implicit or unconscious bias can have implications for health. Researchers have posited that the external classification of race and ethnicity, particularly, classification as a member of a group that has historically been the target of oppression, exploitation, and negative stereotypes (i.e., blacks, Latinxs and Native Americans) may serve as the basis for unfair treatment or differential access to opportunities and resources that are important to maintain health [21]. For example, being socially assigned as black or Latinx may expose individuals to unique psychosocial stressors, such as racial discrimination, that are associated with poorer health outcomes [4, 20]. Moreover, there is a growing body of research that uses socially-assigned race to examine the health, social, and economic implications of being perceived as white (or as a member of a dominant social group) in comparison to those who are socially-assigned as non-white or a non-dominant group [20, 22–25]. This review represents to our knowledge, the first systematic and comprehensive assessment of the research on socially assigned race and its relationship with health and health-related outcomes.

Examining socially-assigned race may provide additional insight into mechanisms that shape population health and reinforce racial/ethnic health inequities. We conducted a scoping review of the peer-reviewed literature to: 1) appraise the evidence on socially-assigned race and health; 2) discuss conceptual and methodological considerations for utilizing socially-assigned race in health-related research; and 3) and identify priorities for future scholarship.

## Materials and methods

This scoping review followed the methodology as described in Preferred Reporting Items for Systematic Reviews and Meta-Analyses extension for scoping reviews (PRISMA-ScR) and adheres to guidelines for a scoping review protocol set out by Arksey and O’Malley to: 1) identify the research; 2) identify the relevant studies; 3) select the studies; 4) chart the data; and 5) collate, summarize, and report the results [26–28].

### Research question

The following research questions were formulated to guide the scoping review:

1. What are the characteristics, contexts, and results of research examining socially-assigned race on health and health-related outcomes?
2. What are the conceptual and methodological gaps in the way socially-assigned race is conceptualized and analyzed in the health literature?

### Information sources and search strategy

Search strategies were developed by the public health librarian (NT) using controlled vocabulary and free-text terms combining two main concepts: (1) socially assigned race and (2) health outcomes (Additional file 1). The published literature was systematically searched in PubMed, Web of Science, EBSCO, and Proquest (Additional file 2). Due to the low number of results in our preliminary searches, we expanded our search to all subscribed databases within the EBSCO and Proquest platforms using the “Choose databases” and “Change databases” features respectively. The search strategy was inclusive of all countries, years of publication, and study designs (qualitative, quantitative, or mixed-methods). Searches were limited to English-language studies published in the peer-review literature through May 2019 and updated on September 27, 2019. The WHO Global Health Library, Bielefeld Academic Search Engine (BASE), Directory of Open Access Journals (DOAJ), MedNar search engine, OpenGrey, EThOS (British Library), and Google Scholar were searched for grey literature and unpublished reports. Reference lists of relevant grey literature, as well of the included studies were hand-searched to identify additional studies. Colleagues and researchers in relevant fields were contacted for assistance in identifying additional studies. Peer-reviewed journal articles were included if (1) socially-assigned race was the primary ‘exposure’ of interest in the analysis and (2) socially-assigned race and the association with either a health, healthcare, or health-related outcome was examined. Studies that included socially-assigned race as a covariate or control variable were excluded.

### Study selection process

The final search results were exported into EndNote and duplicates were removed. Each title and abstract was screened for inclusion by two independent reviewers (JAL, KW). The full-text of the remaining articles were screened independently by two reviewers (JAL, KW) to determine if the inclusion criteria were met. If there were any discrepancies that arose between the two reviewers, a third reviewer (JC) was consulted to resolve the disagreement by consensus.

### Charting the data and reporting the results

The study team developed a data-charting form to determine the appropriate variables to extract and document the relevant study characteristics. Two reviewers (JAL, KW) independently charted the data and discussed the results. The articles included in the sample were characterized by article identifiers (e.g., authors, year of publication), study identifiers (e.g., country, sample size, study design), study sample characteristics (e.g., N, age, sex, race/ethnicity), purpose of study, socially-assigned race/ethnicity measure, outcome, and result by narrative format. Tables were created to reflect the studies included, study designs, publication years and key characteristics of the study populations. An assessment of the quality of the studies was not performed, which is in alignment with scoping review methodology.

## Results

**Fig. 1:**
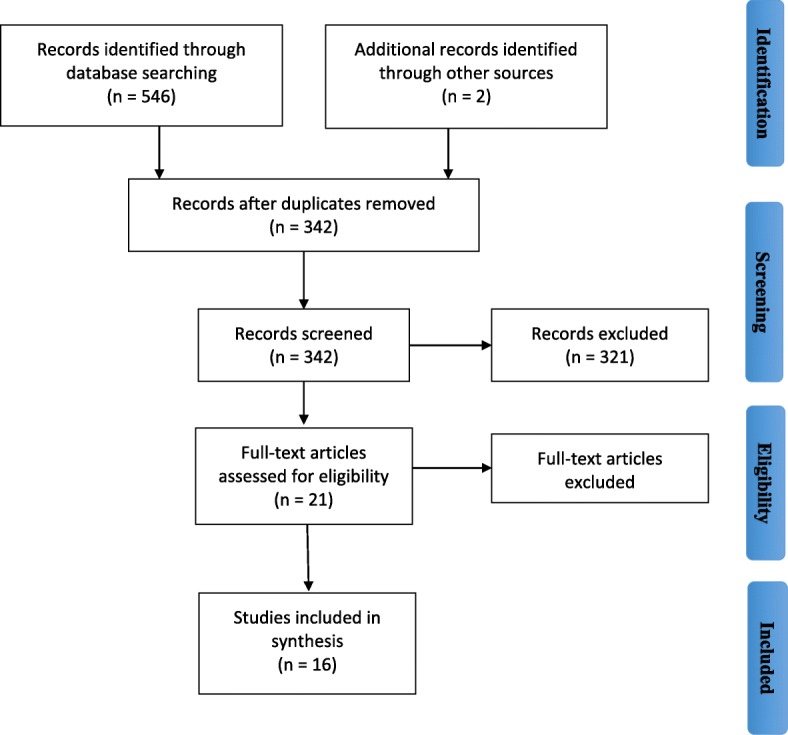
Search process illustrated in a PRISMA flowchart for scoping review

The initial search in May 2019 and the updated searches in September 2019 yielded a total of 548 items. After exclusion of duplicates (*n* = 206), the remaining 342 articles were screened independently by two reviewers (JAL, KW) to determine if the inclusion criteria were met. Three hundred twenty-one articles were excluded for not meeting the inclusion criteria and three articles were excluded after full text review (JAL, KW), leaving 18 for inclusion in the review. The study selection process is illustrated in Fig. 1. There were 12 studies conducted in the United States, 4 in New Zealand, 1 in Canada, and 1 in Latin America (which comprised of several countries including Brazil, Colombia, Mexico, and Peru). The majority of included studies were cross-sectional in design and constituted empirical quantitative analyses, with the exception of one qualitative analysis. A summary description of each study is presented in Table 1.

**Table 1:** Summary description of articles^a^ included in review by health outcome

	Reference name	Dataset	Sample size, and study participant age and sex	Socially-assigned race measurement	Health outcome (s)	Racial/ethnic groups included
Self-rated health and physical health outcomes	Jones et al. 2008 [20]	Behavioral Risk Factor Surveillance System 2004	*N* = 34,755 18+	How do other people usually classify you in this country [United States]?	Self-rated health	American Indian / Alaskan Native, Asian, Black, Hispanic, More than one race, Native Hawaiian or other Pacific Islander, Other, White
	Veenstra 2011 [29]	Survey Research Center at the University of Victoria 2009	*N* = 1499 18+	“And what about other people who you meet, what racial back-ground do other people tend to think you are? Do they think that you’re White, Asian, South Asian, Black, Southeast Asian, or Aboriginal, or perhaps some combination of these, or maybe something else I haven’t mentioned?”	Self-rated health, Hypertension	Aboriginal, Asian, Black, Other, South Asian, Southeast Asian, White
	Cormack et al. 2013 [25]	New Zealand Health Survey 2006–2007	*N* = 12,488 15+	“How do other people usually classify you in New Zealand?”	Self-rated health	Asian, European ethnic group, Māori, Other ethnic group, Pacific
	Harris et al. 2013 [30]	New Zealand Health Survey 2006–2007	*N* = 3160 15+	“How do other people usually classify you in New Zealand?”	Self-rated health	Māori
	Perreira et al. 2014 [31]	Project on Ethnicity and Race in Latin America (PERLA) 2010	*N* = 4921 18–65	Racial category assigned by interviewer	Self-rated health	Indigena, Mestizo, Mulato or Black, Other, White
	Vargas et al. 2015 [11]	Latino Decisions/impreMedia 2011	*N* = 1200 18+	“How do other people usually classify you in the United States?	Self-rated health	Latinx
	Garcia et al. 2015 [6]	Latino Decisions/impreMedia 2011	N = 1200 18+	How do other people usually classify you in the United States?	Self-rated health	Latinx
	Cobb et al. 2016 [13]	Nashville Stress and Health Study 2011–2014	*N* = 1186 22–69	Socially-assigned skin tone (proxy for socially-assigned race) assessed by interviewers	Allostatic load	Black, White
	Muriwai et al. 2016 [32]	New Zealand Attitudes and Values Study (NZAVS) - Maori Focus questionnaire 2012	*N* = 667 18+	Perceived appearance	Smoking status	Māori
	Lopez et al. 2018 [33]	Latino National Health and Immigration Survey (LNHIS) 2015	*N* = 1197 18+	How do other people usually classify you in the United States? What is your street race?	Self-rated overall physical health	Latinx
	Lawrence et al. 2019 [34]	Arizona Behavioral Risk Factor Surveillance System 2013–2014	*N* = 8370 18+	How do other people usually classify you in this country [United States]?	Self-reported diabetes	Latinx, White
Preventive health screenings	MacIntosh et al. 2013 [23]	Behavioral Risk Factor Surveillance System 2004	*N* = 33,679 18+	How do other people usually classify you in this country [United States]?	Receipt of influenza vaccination (> 65); receipt of pneumococcal vaccination (> 65); Breast cancer screening (women, > 40); Cervical cancer screening in last 3 years (women, > 21); Prostate cancer screening (PSA and DRE, men > 50); Colorectal screening (FOBT, colonoscopy, > 50)	American Indian / Alaskan Native, Asian, Black, Hispanic, More than one race, Native Hawaiian or other Pacific Islander, Other, White *responses other than non-Hispanic white were categorized as minority
	Saperstein 2009 [35]	National Survey of Family Growth 1988	*N* = 8450 15–44 Women only	Racial category assigned by an observer (the interviewer)	Receipt of pap smear last 12 months; receipt of breast exam; blood pressure checked	Black, White, other
Mental health outcomes	Campbell and Troyer 2007 [7]	National Longitudinal Survey of Adolescent Health (Add Health) 1994–2002	*N* = 436 18–28	Racial category assigned by an observer (the interviewer)	Depressive symptoms, suicidal ideation, suicidal attempts, fatalism, use of psychological counseling	American Indian, Latinx, White, Black, Asian
	Veenstra 2011 [29]	Survey Research Center at the University of Victoria 2009	*N* = 1499 18+	“And what about other people who you meet, what racial back-ground do other people tend to think you are? Do they think that you’re White, Asian, South Asian, Black, Southeast Asian, or Aboriginal, or perhaps some combination of these, or maybe something else I haven’t mentioned?”	Depressive feelings, self-rated mental health	Aboriginal, Asian, Black, Other, South Asian, Southeast Asian, White
	Cormack et al. 2013 [25]	New Zealand Health Survey 2006–2007	N = 12,488 15+	“How do other people usually classify you in New Zealand?”	Psychological distress	Māori, Pacific peoples, Asian, European/other ethnic groups
	Harris et al. 2013 [30]	New Zealand Health Survey 2006–2007	N = 3160 15+	“How do other people usually classify you in New Zealand?”	Psychological distress	Māori
	Pirtle and Brown 2016 [36]	National Longitudinal Survey of Adolescent Health (Add Health) Wave 1 (1994–1995) and Wave 3 (2001–2002)	*N* = 813 13+	Racial category assigned by an observer (the interviewer)	Depressive symptoms, suicidal ideation, use of psychological counseling	American Indian
	Saperstein et al. 2016 [15]	National Longitudinal Survey of Adolescent Health (Add Health) Wave 3 (2001–2002) and Wave 4 (2007–2009)	N = 12,817 24–32	Racial category assigned by an observer (the interviewer)	Depressive symptoms	Asian, Black, Native American, White
	Lopez et al. 2018 [31]	Latino National Health and Immigration Survey (LNHIS) 2015	N = 1197 18+	How do other people usually classify you in the United States? What is your street race?	Self-rated overall mental health	Latinx
Health services utilization and engagement	MacIntosh et al. 2013 [23]	Behavioral Risk Factor Surveillance System 2004	N = 33,679 18+	How do other people usually classify you in this country [United States]?	Have a personal physician	American Indian / Alaskan Native, Asian, Black, Hispanic, More than one race, Native Hawaiian or other Pacific Islander, Other, White *responses other than non-Hispanic white were categorized as minority
	Reid et al. 2016 [37]	Hauora Manawa/Heart Study: The Community Heart Study 2008–2009	*N* = 40 25–64	Perceived appearance	Experiences with accessing and engaging with primary health care professionals	Māori
Healthcare discrimination	MacIntosh et al. 2013 [23]	Behavioral Risk Factor Surveillance System 2004	N = 33,679 18+	How do other people usually classify you in this country [United States]?	Perceived healthcare discrimination	American Indian / Alaskan Native, Asian, Black, Hispanic, More than one race, Native Hawaiian or other Pacific Islander, Other, White *responses other than non-Hispanic white were categorized as minority
	Cormack et al. 2013 [25]	New Zealand Health Survey 2006–2007	N = 12,488 15+	“How do other people usually classify you in New Zealand?”	Ever experience discrimination in health	Māori, Pacific peoples, Asian, European/other ethnic groups
	Harris et al. 2013 [30]	New Zealand Health Survey 2006–2007	N = 3160 15+	“How do other people usually classify you in New Zealand?”	Ever experience discrimination in health	Māori
	Stepanikova and Oates 2016 [24]	Behavioral Risk Factor Surveillance System 2004–2013	*N* = 113,212 18+	How do other people usually classify you in this country [United States]?	Perceived discrimination in health care	Asian, Black, Hispanic, Native American, Other, White

^a^The number of entries in the table is more than the total number of articles found in the review since an article may have had more than one outcome

### Measurement of socially-assigned race

Studies operationalized socially-assigned race either as perceived (the race one believes others assume you to be) or observed (by an interviewer based on observable characteristics). The majority of studies ascertained perceived socially-assigned race by respondents’ answers to the question: “*How do other people usually classify you in this country*?” [6, 11, 20, 23–25, 30, 33, 34]. In the United States, this question is most frequently collected via the Behavioral Risk Factor Surveillance System (BRFSS). BRFSS is an annual state-level, telephone survey of non-institutionalized adults living in the United States that monitors health behaviors, chronic health conditions, and utilization of preventive health services. The socially-assigned race question is collected via an optional module or included as a state-added question among all racial/ethnic groups the survey is administered to. Other studies conducted in the United States examining socially-assigned race that focused specifically on the experiences of Latinx populations, utilized the 2011 Latino Decisions/ImpreMedia survey. This survey is nationally representative of Latinxs and is designed to ascertain information about attitudes and experiences of health, health care, policy, discrimination, and detailed information on national origin, nativity, acculturation, and citizenship. In New Zealand, socially assigned race was collected during the administration of the New Zealand Health Survey (NZHS; 2006–2007). NZHS collects self-reported data from individuals aged 15+ on physical and mental health and health service use. In Veenstra [29] the question about socially-assigned race was queried among Canadians living in Vancouver and Toronto as: “… And what about other people who you meet, what racial background do other people tend to think you are? Do they think that you’re white, Asian, South Asian, Black, Southeast Asian, or Aboriginal, or perhaps some combination of these, or maybe something else I haven’t mentioned?“ [29].

In several studies, socially-assigned race was observed, where the interviewer classified participants’ race/ethnicity or skin tone (as a proxy for race) [7, 12, 15, 31, 36]. Several studies utilized data from the National Longitudinal Study of Adolescent Health to Adults (Add Health). In Add Health, at the end of the interview, interviewers were asked questions about the participant including assigning the participant to a racial category based on the interviewer’s observation [7]. Perceived appearance, one’s own subjective evaluation of their appearance, was used to capture socially-assigned race in two studies [32, 37]. In another study, the interviewer’s rating of skin tone was used as a marker of socially-assigned race for its “generalized perception of other” [13]. Lopez et al. uses a slightly different approach to measure socially-assigned (i.e., ascribed) race by querying respondents about self-perceived race, socially-ascribed race and street race [33]. The socially-ascribed race was asked similarly to the question on the BRFSS. The street race question asked: “If you were walking down the street, what race do you think other Americans who do not know you personally would assume you were based on what you look like?” There were five street race categories including: white, Latinx, black, Arab, and Mexican.

### Patterns of socially-assigned race by race/ethnicity

In the United States, the collection of socially-assigned race using data from the BRFSS is inclusive of multiple racial/ethnic groups and multiracial individuals. Research has shown that congruence between self-identified and socially-assigned (concordance) appears to be greatest among individuals that self-identify as white or black (98.4 and 96.3% respectively.) [20] Disagreement between self-identification and socially-assigned race (discordance), occurs most frequently among Latinx, AI/AN, NHOPI, and multiracial groups [14, 20–22]. For example, Jones et al. demonstrated that among those who self-identify as Latinx, 63.0% were socially-assigned as Latinx, 26.8% white and 3.5% black [20]. Among American Indian, 47.6% were classified by others as white and for NHOPIs, 35.1% were socially assigned as white [20].

Socially-assigned race studies conducted in New Zealand, included Māori, Pacific peoples, Asian and European ethnic groups [25]. In this context, concordance between self-identified and socially assigned race was greatest among individuals who self-identified as European (97.6%, which is considered the dominant ethnic group) and Asians (92.7%). Discordance was highest among individuals who self-identified as Māori, Pacific, and those who identified as multi-ethnic [25]. In a study conducted across several Latin American countries, interviewers reported their assessment of respondent’s race/ethnicity according to the following 5 categories: white (blanca/branca); mixed-white (mestizo/parda), mixed-black (mulato), indigenous (indigena), and black (negra/preta) or other [31]. It was reported that interviewers’ classifications of respondents matched respondents’ self-classifications in 61% of cases [31]. In a study with respondents from Vancouver and Toronto Canada, a lower percentage of Black (73.9%) and South Asian (65.7%) respondents reported a greater mismatch between self-identified and socially-assigned race in comparison to Whites (95.9%) and Asians (91.3%) [29].

### Health outcomes

#### Self-rated health and physical health outcomes

The majority of studies examined the relationship between socially-assigned race (and level of agreement between self-identified and socially-assigned race) with self-rated health [6, 11, 20, 25, 29, 30, 33]. Overall, results varied, with half of the studies (5 out of 7) demonstrating that social assignment as white (or European) was beneficial and associated with increased reports of excellent or very good self-rated health – regardless of self-identification [11, 20, 25, 29, 30]. The other remaining studies found null associations [6, 11, 33]. Results from one study demonstrated that the relationship between socially-assigned race and self-rated health among Latinxs who were socially-assigned as Latinx was dependent upon classification of citizenship status, nativity and national origin [11]. When the analyses were not disaggregated by these factors, there was no relationship between socially-assigned race and self-rated health. Few studies examined self-reported physical health outcomes or behaviors such as self-rated diabetes or hypertension or health behavior [29, 34]. In both of these studies, discordance between self-identified and socially-assigned race (as an historically oppressed racial/ethnic group) was associated with higher odds of diabetes and hypertension. Another study, investigated the association between perceived appearance as Māori and smoking status and demonstrated that Māori who were perceived by others as Māori were more likely to be considered smokers in comparison to Māori who were perceived by others as European after adjustment for perceived discrimination and demographics [32]. Only one study used a biomarker of physical health. Cobb et al. [13] investigated whether interviewer observed socially-assigned race was associated with allostatic load, as an indicator of physiologic dysregulation [13]. In the Cobb et al. [13] study, the strength of the association between self-identified versus socially-assigned race was relatively equal, with socially-assigned race showing a slightly higher estimate.

#### Mental health

A total of 7 studies examined mental health outcomes which included measures of self-rated mental health, depressive symptoms, psychological distress, suicidal ideation, suicide attempts, and use of psychological counseling generated mixed results [7, 15, 25, 29, 30, 33, 36]. In 5 of the 7 studies assessing mental health outcomes, a positive association with socially-assigned race and poorer mental health outcomes was detected [8, 25, 29, 30, 36]; however a null relationship was uncovered in one study [33]. Contrarily, in one study, respondents self-identified as white and were interviewer-classified as black had significantly fewer depressive symptoms than respondents who self-identified and were interviewer-classified as black [15]. In studies conducted in New Zealand, persons ascribed as Māori or any other non-European group had higher mean levels of psychological distress in fully adjusted models compared to Europeans socially-assigned as European [25, 30]. Among American Indian adolescents, elevated levels of poorer mental health was associated with discordance between self-identified and observed (interviewer) racial identification [36].

#### Preventive health screenings

Two studies examined preventive health screenings [23, 35]. An interviewer observed measure of socially-assigned race was used to investigate the relationship between self-identified and socially-assigned race and racial differences in reported health screenings among black and white adult women [35]. Saperstein [35] found that socially-assigned race was a stronger predictor of racial differences in breast exams and blood pressure checks than self-identified race. Using a measure of perceived socially-assigned race, MacIntosh et al. [23] examined a comprehensive list of preventive age-appropriate healthcare screenings including receipt of influenza and pneumococcal vaccinations and screenings for breast, cervical, prostate, and colorectal cancers using data from BRFSS. In this study, the relationship between socially-assigned race and preventive health screenings was dependent on the specific health screening assessed. Social assignment as white among self-identified as non-white (e.g., black, Latino, American Indian, or Asian, Pacific Islander, Native Hawaiian, multiracial, or other) was associated with higher odds of receipt of pneumococcal and influenza vaccinations compared to those ascribed as non-white. For prostate and colorectal cancer screenings there were no differences between groups [23]. Respondents who self-identified and were socially-assigned as non-white, were more likely to have the age-appropriate breast and cervical cancer screenings when compared to women who self-identified and were socially-assigned as white [23].

#### Health service utilization and engagement

Two studies examined the relationship between socially-assigned race and health service utilization as measured as having a personal physician and perceived access to and engagement with primary healthcare. In a quantitative study using data from the BRFSS, individuals who self-identified and were socially-assigned as non-white were less likely to report having a personal physician when compared to individuals who self-identified and were socially-assigned as white [23, 37]. A qualitative study was conducted among adult Māori to determine the significance of social-assignment when interacting and seeking healthcare services with non- Māori providers [37]. Results from the thematic analyses of in-depth interviews revealed that social-assignment as New Zealand European was associated with greater ease in acquiring quality healthcare. Exposure to discriminatory treatment and negative stereotypes emerged as salient themes related to social-assignment as Māori [37]. These narrative descriptions provide rich details of individual experiences to accessing healthcare and quality interactions with healthcare professionals that can be further used to generate testable theories and mechanisms linking socially-assigned race and population health.

#### Experiences of healthcare discrimination

The bulk of research examining racial discrimination primarily does so through the lens of self-identification. However, we identified 4 articles that examined experiences of healthcare discrimination by socially-assigned race [23–25, 30]. Cormack et al. [25] and Harris et al. [30] compared the distribution of racial discrimination in health care by socially-assigned race. Both studies found that respondents who were socially assigned as Māori or other non-European ethnicities reported higher levels of discrimination in health [25, 30]. Studies that used the BRFSS also found that reports of healthcare discrimination was highest among non-white respondents who were socially-assigned as non-whites compared with non-white respondents who were socially-assigned as white [23, 24].

## Discussion

There is increasing scholarship examining socially-assigned race and health outcomes. The final synthesis included 18 articles representing a range of health and healthcare-related outcomes. Although this scoping review demonstrates limited evidence with respect to **the** volume of studies, several themes were revealed through the search, data extraction, and analysis stage. These themes have been grouped according to conceptual considerations, methodological issues, and recommendations for future research, which frame the discussion.

### Conceptual considerations

The multidimensionality of race is rooted in theory about reflected appraisals which specifies that an individual’s idea of self is in part derived from social interactions with others [33, 38, 39]. The literature appraised emphasizes a relational dimension of socially-assigned race that identifies “a group’s location within a social hierarchy (e.g., minority versus majority status)” (page 251 [9]) and underscores how this hierarchy differentially affects group well-being and health [40]. Social assignment of race/ethnicity is experienced according to others’ perceptions and in part reflects a racial hierarchy rooted in accrued privilege [14, 25]. The hierarchy confers an advantage to individuals racially classified as white and penalizes those perceived by others as a member of a historically oppressed group (i.e., lower on the racial hierarchy).

The question that arises is whose perceptions are relevant and does this affect the construct validity of a measure of socially-assigned race? When using a measure of perceived socially-assigned race, we rely on the respondent to indicate their perception of how others are likely to ascribe them and surveys seldom inquire about **the** race/ethnicity of the perceiver. An underlying assumption of socially-assigned race is the significance of the classification of members of a higher-status or dominant group. These members typically have greater access to power and resources and have a tendency to reify historical, institutional and systemic inequalities that foster and maintain the power dynamic [16]. However, the racial/ethnic background of the “perceiver” is unknown. Moreover, how one is perceived may vary by the race/ethnicity of the perceiver [14]. To our knowledge, datasets that collect socially-assigned race, do not additionally inquire about the race/ethnicity of the “perceiver” or the situation in which the external classification occurs. Further, it is not clear whether there are certain factors that may influence how one perceives external racial attribution. Vargas and Stainback [14] sought to contextualize factors that influence incongruence between self-identified race and socially-assigned race using data from the Portraits of American Life Study [14]. Their findings suggest that individuals who reported a mismatch (incongruence) between self-identity and social-assignment were more likely to report lower levels of ethnoracial unity (i.e., feel less close to other members of self-identified racial group) and racial identity salience (i.e., lack of connection with other members of self-identified racial group) in comparison to individuals who were congruent on self-identification and social-assignment [14]. Qualitative research designs may prove to be particularly informative in systematically evaluating these issues which may help improve the construct validity of measures of socially-assigned race.

The health impact of the generalized perception of others may differ by the racial/ethnic groups targeted for racialization [18]. An important conceptual consideration is related to assumptions surrounding classification as a lower-status or “minority” group member. Racial classification reflects physical, socioeconomic, and cultural perceptions of an individual [21]. There may be observed differential impacts on health based on the type of perceived racial classification. In the United States, some have argued that there is a hierarchical system of racial classification that presupposes racial discrimination. For example, Latinx populations may be perceived as white, Latinx, black/African-American. The extent to which patterns in health risk are associated with perceptions as Latinx, versus black/African-American, versus multi-racial group is unclear. It is possible that the health risk may mirror the intraethnic heterogeneity of health outcomes such as diabetes which align with the racial stratification of Latinx groups [41]. More specifically, racial differences in diabetes prevalence are highest among Latinxs who self-identify as black (i.e., Puerto Ricans, Dominicans) in comparison to those who self-identify as white or Latinx [41–43]. Comparisons of these health differences have been under-investigated in large part due to insufficient sample sizes and are worthy of further exploration.

Another conceptual consideration is the choice for reference group. This is particularly applicable in studies exploring level of agreement between self-identified and socially-assigned race. The majority of studies in this review, that were conducted in the United States, used self-identified non-Hispanic whites who were socially-assigned as non-Hispanic whites as a referent group. This choice of reference is theoretically relevant for studies probing the health advantage of being perceived as white. However, alternative choices for referent groups, for example, being self-identified and socially-assigned as a non-white racial/ethnic group have also been employed in studies to facilitate interpretation of the outcome [23].

### Methodological issues

There are several methodological issues related to study design, data availability and analytic strategies that deserve further attention. The majority of studies assessed were quantitative. However, employing qualitative or mixed-methods research designs would be an important contribution to further elucidate the mechanisms underpinning socially-assigned race and health. Utilizing these designs has the potential to gain in-depth understanding of one’s lived experience that may help to generate robust theories and elucidate pathways through which social-assignment is related to health. Further, the detail information obtained from qualitative techniques could be useful for informing the interpretation and corroboration of quantitative data. Additionally, qualitative techniques that combine innovative approaches such as the use of multimedia vignettes or simulated and virtual reality platforms may be used to assess the scope of bias due to socially-assigned race among health care providers.

The availability of data sets collecting and documenting socially-assigned race poses a challenge to generating future research investigating socially-assigned race and health. Overall, the studies were cross-sectional, with longitudinal investigations remaining unexplored. Large population-based surveys such as the BRFSS collects data on socially-assigned race. However, the use of this survey has been limited because states opt-in for the collection of this data. Beginning in 2002, selected states included the *Reactions to Race* module, which also asked questions about race consciousness, emotional and physical reactions to race-based treatment, and perceived differential treatment in employment and healthcare settings. The *Reactions to Race* module is not included as part of the core component of the BRFSS questionnaire that comprises a set of standard questions asked by each state each year. Instead, it has been considered an optional module, where states make the choice to adopt the modules to be administered for a given year. Stepanikova et al. [24] used socially-assigned race collected in BRFSS by pooling data across years (2004–2013) and 17 states and the District of Columbia to yield a large sample size [24]. In recent years, some of the questions from the BRFSS *Reactions to Race* module were included as ‘state-added’ questions - when an individual state elects to include questions of their choosing that may include a subset or a single question from an optional module or validated scale. Inclusion of state-added questions are not reported on the main BRFSS website and can be only be determined by reviewing each individual state’s BRFSS data documentation. This presents a challenge, because it is difficult to determine the extent to which states are administering the socially-assigned race question or other *Reactions to Race* module questions.

The utility of socially-assigned race in BRFSS has been critiqued for its lack of representativeness, particularly for national Latinx populations [11]. Because of the limited number of states that administered the *Reactions to Race* module, it largely reflects Latinx populations that are predominantly Mexican, whereas states that have higher concentrations of Puerto Ricans, Dominicans, Central Americans, and Cubans have not well represented. Surveys such as the 2006 Portraits of American Life, the 2011 Latino Decisions/ImpreMedia, and the Latino National Health and Immigrant Survey include samples that are intended to be more representative of the Latinx population in comparison to the BRFSS data. These surveys have the capacity to explore differences in socially-assigned race by finer delineations of national origin, acculturation, and citizenship. While these data sets are ideal to answer questions and understand socially-assigned among Latinx populations, the data sets tend to have smaller sample sizes, are not conducted in consecutive years, and collect limited health outcome data in comparison to the BRFSS.

The socially-assigned race literature can benefit from the extension and focus on other racial/ethnic groups or historically oppressed populations. However, the issue of sufficient sample size is a major challenge for examining socially-assigned race in other racial/ethnic groups such as Native Americans, Native Hawaiians and Other Pacific Islanders, multi-racial, or indigenous populations. A study that was conducted using data from Vancouver and Toronto, Canada could not analyze data for mismatches between self-identified and socially-assigned race for Aboriginal and Southeast Asian populations [29]. Further, we identified a dearth of data sets that contain socially-assigned race measures data on adolescents. One study used data from Add Health to examine the link between socially-assigned race and health among Native American and white adolescents [36]. However, in our review of the literature this was the only study conducted among adolescents.

Future socially-assigned race research would benefit from theoretically driven analytical considerations related to model specification. Many of the included studies are minimally adjusted for potential confounders. Included studies have assessed the association between socially-assigned race and self-reported health without adjusting for behavioral factors and health characteristics (i.e. physical activity, smoking, BMI, fruit/vegetable intake) which have been documented to be associated with self-rated health. Additionally, using measures of citizenship status, nativity, and time in the U.S. as potential effect modifiers may help clarify some of the observed health patterns among Latinx populations. In addition to including other health-related covariates in analyses and conducting more detailed assessments, we posit that coping styles may also differ by ascribed race and should be examined.

The majority of studies examined the main effects of socially-assigned race on health outcomes. Further explanation of mechanisms and potential effect moderators by the relationship between socially-assigned race and health may afford a theoretical foundation to disentangle processes that influence racialization and subsequent inequities in health and healthcare. Most studies included measures of socioeconomic status, such as household income, educational attainment and occupational status as mediators of the association between socially-assigned race and health. Few studies have yet to test the suggested mechanisms through which socially-assigned race is posited to operate such as exposure to individual-level discrimination. Moreover, potential variables identified as effect modifiers, such as neighborhood racial-ethnic residential segregation or stress buffers (e.g., vigilance and anticipatory stress) that may diminish or amplify health effects, have rarely been explored.

### Recommendations for future research

Although, our knowledge of racial health inequalities is predominantly ascertained from studies that measure self-identified race/ethnicity, we see great utility in incorporating measures of socially-assigned race in population health studies. It is imperative that we advocate for and include questions about socially-assigned race in addition to other multidimensional measures of race in representative population-based datasets. The incongruence between self-identified and socially-assigned race can help in answering questions related to the persistence and maintenance of racial health inequalities that warrant further empirical investigation. Towards this end, there is a real need for inclusive race/ethnicity data collection efforts in our public health monitoring and surveillance systems and surveys to move closer to achieving health equity.

Use of a single, unidimensional measure of race does not provide sufficient detail about intraracial processes of racialization and health. Studies determining the explanatory power that socially-assigned race has in differentiating intraracial experiences of race and racism and subsequent variations in health are needed. We have a minimal understanding of the extent to which socially-assigned race captures variations in population health. There are increasing efforts to disaggregate the health status of Latinx populations according to foreign-born status and country of origin to capture additional variation in health profiles. However, fewer studies capture racial heterogeneity among Latinx population by using a measure of socially-assigned race to broaden our knowledge regarding Latinx health inequalities. For example, the patterns of health risk and advantage have not been fully explored among Latinxs who are socially-assigned either as Latinx, Black, white, or some other racial/ethnic group. Socially-assigned race-specific reporting of health may uncover variations that are obscured by the use of self-identified race. There are some findings which suggest that educational and economic profiles also vary by socially-assigned race and we know from prior research on self-identified race and health, that these factors are part of the pathway through which “race” influences health [44].

Additional research that considers how measuring socially-assigned race affects population health disparities estimates are warranted and have the potential to provide greater insight into the health consequences of the social construction of race and potential targets for social and policy approaches to address inequality. The findings also call attention to the magnitude of racial/ethnic health and healthcare inequalities and how these estimates may be affected by the way race/ethnicity is collected and measured [8, 35]. Saperstein [35] compared the association between interviewer-classified race and self-identified race to receipt of various health screenings (e.g., pap smear, blood pressure, and breast exam) among women [35]. The results suggested that interviewer-classified race, as compared to self-identified race, was a stronger predictor of racial differences in health screenings. A study that used data from BRFSS found that self-identified and socially-assigned race were both independently associated with perceived discrimination in health care [24]. The findings from this study revealed that socially-assigned race was a better predictor of perceived discrimination in health care in comparison to self-identified race. Another study evaluated self-reported race/ethnicity and interviewer-ascribed race/ethnicity and income inequality in Brazil [45]. The magnitude of association between interviewer-ascribed race/ethnicity and income inequality was larger when compared to a measure of self-reported race/ethnicity. These studies illustrate the importance of considering multiple dimensions of race. In the aforementioned examples, the mechanisms of inequality were best represented by a measure of socially-assigned race. The operationalization of race such that it is ascribed by someone else may resemble racial discrimination and implicit bias and could lead to a more appropriate estimation of the magnitude of disparities. These examples also show how the reliance upon a single measure of race, namely self-identified race/ethnicity, can underestimate the level of health inequities. However, it is not clear the extent to which one measure of race may be more (or less) strongly associated with health or factors that influence health. It is possible that the relative strength of self-identified versus socially-assigned race varies by health outcomes and may be a function of theoretically distinct mechanisms that are responsible for the health disparity. Saperstein [35] demonstrated that socially-assigned race was a stronger predictor of health outcomes that were encounters in health care or clinic settings versus self-identified race which was a stronger predictor of group differences in outcomes. An explanation of this difference is related to the inherent value given to others classification of one’s race and the implicit biases and prejudices that accompany it [35]. Scholars contend that socially-assigned race may be more closely associated with institutionalized racism and experiences of discrimination [29]. Assumed cultural differences and stereotypes about one’s race may be more salient to the quality of interactions with health care providers, receipt of a health screening during a medical visit, or receipt of pain medication in an emergency room, more so than self-identified race. Future lines of inquiry would substantially benefit from the thoughtful, theory-driven selection of a specific dimension of race and health outcomes since different measures of race may offer different explanations or have different implications for addressing the inequality [35].

The social construction of race/ethnicity and racial hierarchies around the world varies depending on the social, historical, and political context of an area. Though substantial work has contributed to our understanding of the racialization process, vis-à-vis socially-assigned race and health, much of this work comes from a United States perspective and has been largely conducted among Latinx populations. Though there are few studies on Native Americans, it is not clear to what extent the health status of members of other racial/ethnic groups are differentially affected by socially-assigned race. Exploring socially-assigned race in other areas (e.g., Europe) with increasing ethnic diversity would be informative. While the implications of socially-assigned race extend beyond the United States to other regions (e.g., Latin America) and countries (e.g., New Zealand) around the world, we need to be cautious about extrapolating findings from one area to another. Research could benefit from a deeper understanding of the process of racialization among other non-dominant racial/ethnic groups or indigenous peoples outside of the United States context. Relatedly, new areas of research interest may entail exploring an expanded set of health care and physiological health outcomes for a more comprehensive picture of health. Moreover, new technologies (e.g., machine learning, automation, algorithms, and phenotype recognition) may influence social assignment of race. While there is evidence regarding the impact of these technologies on racial bias and profiling, the potential impact on health and healthcare related outcomes is unknown and warrants future study. Given the substantial gaps in qualitative and quantitative data collection on socially-assigned race, creative ways to link existing datasets with big data sources and use of innovative qualitative techniques may provide opportunities to generate new insights and a comprehensive understanding of the relationship between socially-assigned race and population health across various contexts. Future scholarship that includes socially-assigned race as a variable to measure and monitor population-level health status and track the racialized experiences of historically oppressed, marginalized, and indigenous groups around the world is a crucial next step for population health inequalities research.

## Conclusions

The results of this scoping review highlight the need to collect, assess, and examine socially-assigned race in research pertaining to racial/ethnic health inequities. The results of the studies in this review tend to reveal a lower quality of health for those who were socially-assigned to a lower-status group as compared to those who are socially-assigned to a higher-status or dominant group. The social-assignment of race may be an additional tool and sentinel indicator to effectively demonstrate how the social construction of race drives differences in health and health care in racialized societies. However, the dearth of data sets containing a multidimensional measures of race in general, and socially-assigned race, more specifically is a challenge and limits the extent to which we can fully understand its impact along finer delineations by various health outcomes, age, and multiple racial/ethnic groupings. Our analysis makes several contributions to the literature by providing insight into the limits of using self-identified race and revealing that socially-assigned race matters in shaping variations in population health. The research on socially-assigned race complements the extant research on the role of racial discrimination. It is becoming increasingly important to leverage data that captures the multiple dimensions of race on the lived experience in a racialized society given shifting population demographics. Improved contextualization of race/ethnicity, specifically characterizing socially-assigned race, may facilitate the examination of more nuanced patterns of racial health advantage and disadvantage which has significant implications for efforts towards addressing persistent racial/ethnic health inequalities.

## Supplementary information

**Additional file 1.** Search terms used in database searches

**Additional file 2.** Search strategy for databases

## Data Availability

All data generated or analyzed during this study are included in this published article and its supplementary information files.
