# Supplementary material for: Socially-assigned race and health: a scoping review with global implications for population health equity
Source: Int J Equity Health. 2020 Feb 10;19:25. doi: 10.1186/s12939-020-1137-5 (PMC7011480; doi:10.1186/s12939-020-1137-5)
Supplement: Supplementary file 1 — Additional file 1. Search terms used in database searches [file 12939_2020_1137_MOESM1_ESM.docx]

**Additional File 1: Search terms used in database searches**

| #1  Socially assigned race | #2  Health outcome |
| --- | --- |
| (expressed OR reflected) AND racial identit* | "Outcome Assessment (Health Care)"[Mesh] |
| ascribed rac* | ethnic disparities |
| colorism | health advantage |
| colourism | health disparities |
| interviewer ascribed | health equity |
| interviewer assigned | health outcomes |
| observer ascribed | health risk |
| observer assigned | health status disparities[Mesh] |
| phenotypic classification | racial disparities |
| racial appraisal |  |
| racial attribution |  |
| racial contestation |  |
| socially-assigned ethnicity |  |
| socially-assigned race |  |
