# Supplementary material for: Socially-assigned race and health: a scoping review with global implications for population health equity
Source: Int J Equity Health. 2020 Feb 10;19:25. doi: 10.1186/s12939-020-1137-5 (PMC7011480; doi:10.1186/s12939-020-1137-5)
Supplement: Supplementary file 2 — Additional file 2. Search strategy for databases [file 12939_2020_1137_MOESM2_ESM.docx]

**Additional File 2: Search strategy for databases**

**Pubmed database**

Search on May 15^th^ 2019; updated on September 27, 2019

| Search | Query | Items found |
| --- | --- | --- |
| #1 | (expressed OR reflected) AND racial identit*) OR "ascribed rac*" OR colorism OR colourism OR "interviewer ascribed" OR "interviewer assigned" OR "observer ascribed" OR "observer assigned" OR "phenotypic classification" OR "racial appraisal" OR "racial attribution" OR "racial contestation" OR "socially-assigned ethnicity" OR "socially-assigned race" | 627 |
| #2 | "Outcome Assessment (Health Care)"[MeSH Terms]) OR (health status disparities[MeSH Terms]) OR "ethnic disparities" OR "health advantage" OR "health disparities" OR "health equity" OR "health outcomes" OR "health risk" OR "racial disparities" | 1,124,379 |
| #1 AND #2 | (expressed OR reflected) AND racial identit*) OR "ascribed rac*" OR colorism OR colourism OR "interviewer ascribed" OR "interviewer assigned" OR "observer ascribed" OR "observer assigned" OR "phenotypic classification" OR "racial appraisal" OR "racial attribution" OR "racial contestation" OR "socially-assigned ethnicity" OR "socially-assigned race"))) AND ((("Outcome Assessment (Health Care)"[MeSH Terms]) OR (health status disparities[MeSH Terms]) OR "ethnic disparities" OR "health advantage" OR "health disparities" OR "health equity" OR "health outcomes" OR "health risk" OR "racial disparities" | **66** |

**Web of Science**

Search on May 15^th^, 2019; updated on September 27, 2019

| Search | Query | Items found |
| --- | --- | --- |
| #1 | TS=(((expressed OR reflected) AND racial identit*) OR "ascribed rac*" OR colorism OR colourism OR "interviewer ascribed" OR "interviewer assigned" OR "observer ascribed" OR "observer assigned" OR "phenotypic classification" OR "racial appraisal" OR "racial attribution" OR "racial contestation" OR "socially-assigned ethnicity" OR "socially-assigned race") | 1,342 |
| #2 | TS=("health status disparities" OR "ethnic disparities" OR "health advantage" OR "health disparities" OR "health equity" OR "health outcomes" OR "health risk" OR "racial disparities") | 99,880 |
| #1 AND #2 | TS=(((expressed OR reflected) AND racial identit*) OR "ascribed rac*" OR colorism OR colourism OR "interviewer ascribed" OR "interviewer assigned" OR "observer ascribed" OR "observer assigned" OR "phenotypic classification" OR "racial appraisal" OR "racial attribution" OR "racial contestation" OR "socially-assigned ethnicity" OR "socially-assigned race") AND TS=("health status disparities" OR "ethnic disparities" OR "health advantage" OR "health disparities" OR "health equity" OR "health outcomes" OR "health risk" OR "racial disparities") | **43** |

**Web of Science Core Collection** includes the following Citation Indexes:

- Science Citation Index Expanded (SCI-EXPANDED) --1945-present
- Social Sciences Citation Index (SSCI) --1956-present
- Arts & Humanities Citation Index (A&HCI) --1996-present
- Conference Proceedings Citation Index- Science (CPCI-S) --1990-present
- Conference Proceedings Citation Index- Social Science & Humanities (CPCI-SSH) --1990-present
- Book Citation Index– Science (BKCI-S) --2005-present
- Book Citation Index– Social Sciences & Humanities (BKCI-SSH) --2005-present
- Emerging Sources Citation Index (ESCI) --2015-present

**EBSCO databases**

Search on May 15^th^ 2019; updated on September 27, 2019

| ((expressed OR reflected) AND racial identit*) OR "ascribed rac*" OR colorism OR colourism OR "interviewer ascribed" OR "interviewer assigned" OR "observer ascribed" OR "observer assigned" OR "phenotypic classification" OR "racial appraisal" OR "racial attribution" OR "racial contestation" OR "socially-assigned ethnicity" OR "socially-assigned race" |
| --- |
| **AND** |
| "health status disparities" OR "ethnic disparities" OR "health advantage" OR "health disparities" OR "health equity" OR "health outcomes" OR "health risk" OR "racial disparities" |

To search all EBSCO databases simultaneously, the search was performed using the “Choose Databases” filter.


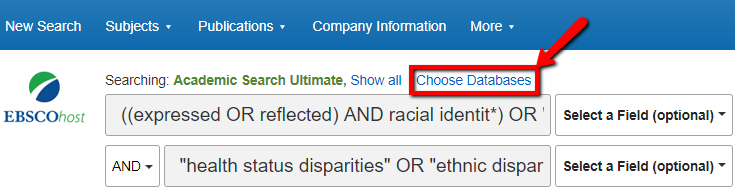


|  | EBSCO Databases | Items found |
| --- | --- | --- |
| 1 | Academic Search Ultimate | 34 |
| 2 | ERIC | 28 |
| 3 | SocINDEX with Full Text | 27 |
| 4 | Education Source | 25 |
| 5 | PsycINFO | 24 |
| 6 | MEDLINE | 22 |
| 7 | Criminal Justice Abstracts | 13 |
| 8 | Race Relations Abstracts | 9 |
| 9 | CINAHL | 6 |
| 10 | CINAHL Plus with Full Text | 6 |
| 11 | Business Source Complete | 5 |
| 12 | Family Studies Abstracts | 5 |
| 13 | Health Source: Nursing/Academic Edition | 5 |
| 14 | MasterFILE Premier | 5 |
| 15 | OpenDissertations | 5 |
| 16 | America: History and Life with Full Text | 2 |
| 17 | Communication & Mass Media Complete | 2 |
| 18 | Family & Society Studies Worldwide | 2 |
| 19 | Historical Abstracts with Full Text | 2 |
| 20 | MLA International Bibliography with Full Text | 2 |
| 21 | Professional Development Collection | 2 |
| 22 | Psychology and Behavioral Sciences Collection | 2 |
| 23 | SPORTDiscus with Full Text | 2 |
| 24 | Abstracts in Social Gerontology | 1 |
| 25 | EconLit | 1 |
| 26 | Film & Television Literature Index with Full Text | 1 |
| 27 | LGBT Life with Full Text | 1 |
| 28 | National Criminal Justice Reference Service Abstracts | 1 |
|  | TOTAL | **240** |

**Proquest databases**

Search on May 15^th^ 2019; updated on September 27, 2019

noft(((expressed OR reflected) AND racial identit*) OR "ascribed rac*" OR colorism OR colourism OR "interviewer ascribed" OR "interviewer assigned" OR "observer ascribed" OR "observer assigned" OR "phenotypic classification" OR "racial appraisal" OR "racial attribution" OR "racial contestation" OR "socially-assigned ethnicity" OR "socially-assigned race") **AND** ("health status disparities" OR "ethnic disparities" OR "health advantage" OR "health disparities" OR "health equity" OR "health outcomes" OR "health risk" OR "racial disparities")

noft = Anywhere except full text

To search all Proquest databases simultaneously, the search was performed using the “Choose Databases” filter.


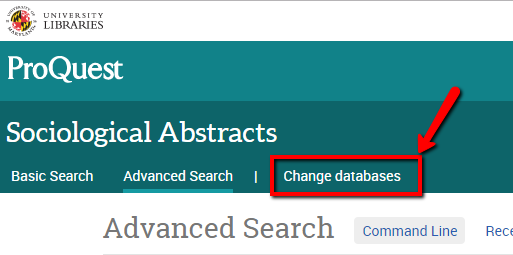


Fifty-five databases were searched simultaneously.


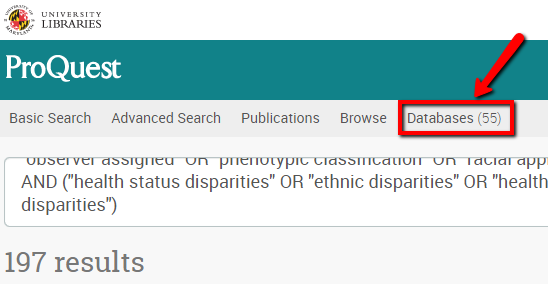


|  | Database | Items found |
| --- | --- | --- |
| 1 | ProQuest Dissertations & Theses Global‎ | 133 |
| 2 | ProQuest Dissertations & Theses Global: Social Sciences‎ | 131 |
| 3 | ProQuest Dissertations & Theses Global: Health & Medicine‎ | 63 |
| 4 | Sociological Abstracts‎ | 37 |
| 5 | Sociological Abstracts‎ | 33 |
| 6 | Ethnic NewsWatch‎ | 17 |
| 7 | Public Health Database‎ | 17 |
| 8 | Social Services Abstracts‎ | 11 |
| 9 | ProQuest Dissertations & Theses Global: History‎ | 11 |
| 10 | ProQuest Dissertations & Theses Global: Literature & Language‎ | 10 |
| 11 | GenderWatch‎ | 9 |
| 12 | ProQuest Dissertations & Theses Global: The Arts‎ | 9 |
| 13 | Criminal Justice Database‎ | 7 |
| 14 | Ebook Central‎ | 7 |
| 15 | Materials Science & Engineering Collection‎ | 5 |
| 16 | Engineering Collection‎ | 5 |
| 17 | PAIS Index‎ | 4 |
| 18 | Engineering Index‎ | 4 |
| 19 | Worldwide Political Science Abstracts‎ | 4 |
| 20 | Engineering Database‎ | 4 |
| 21 | ProQuest Dissertations & Theses Global: Business‎ | 4 |
| 22 | ProQuest Dissertations & Theses Global: Science & Technology‎ | 4 |
| 23 | Materials Science Collection‎ | 2 |
| 24 | Materials Science Database‎ | 2 |
|  | TOTAL | 533 |
|  | *Note:* Proquest database removed the duplicates across its databases when displaying the final set of unique items. | **197** |
